# Delineating the Role of Histamine-1- and -4-Receptors in a Mouse Model of Th2-Dependent Antigen-Specific Skin Inflammation

**DOI:** 10.1371/journal.pone.0087296

**Published:** 2014-02-04

**Authors:** Subhashree Mahapatra, Melanie Albrecht, Barbara Behrens, Adan Jirmo, Georg Behrens, Christina Hartwig, Detlef Neumann, Ulrike Raap, Heike Bähre, Christina Herrick, Anna-Maria Dittrich

**Affiliations:** 1 Department for Pediatric Pneumology, Allergology and Neonatology, Medical School Hannover, Hannover, Germany; 2 Department of Clinical Immunology and Rheumatology, Medical School Hannover, Hannover, Germany; 3 Institute of Pharmacology, Medical School Hannover, Hannover, Germany; 4 Department of Dermatology and Allergy, Medical School Hannover, Hannover, Germany; 5 Department of Dermatology, Yale School of Medicine, New Haven, Connecticut, United States of America; Istituto Superiore di Sanità, Italy

## Abstract

**Background:**

Histamine drives pruritus in allergic skin diseases which clinically constitutes a most disruptive symptom. Skin pathology in allergic skin diseases is crucially influenced by different T-helper subsets. However, the contribution of different histamine-receptors to T-helper cell dependent skin pathology has not been definitively answered. Models which can specifically address the functional role of T-helper subsets and the mediators involved are therefore valuable to gain further insights into molecular pathways which contribute to allergic skin disease. They might also be helpful to probe amendable therapeutic interventions such as histamine-receptor antagonism.

**Objective:**

Establishing an adoptive transfer model for antigen-specific Th cells, we aimed to delineate the role of histamine H_1_- and H_4_-receptors in Th2-dependent skin inflammation.

**Methods:**

*In-vitro* differentiated and OVA primed Th2 cells were adoptively transferred into congenic recipient mice. *In vivo* treatment with specific histamine H_1_- and H_4_-receptor antagonists was performed to analyze the contribution of these histamine-receptors to Th2-dependent skin pathology in our model. Analysis four days after epicutaneous challenge comprised skin histology, flow cytometric detection of transferred T-helper cells and analysis of antigen-cytokine profiles in skin-draining lymph nodes.

**Results:**

Use of specific H_1_- and H_4_-receptor antagonists revealed a crucial role for H_1_- and H_4_-receptors for Th2 migration and cytokine secretion in a Th2-driven model of skin inflammation. While H_1_- and H_4_-receptor antagonists both reduced Th2 recruitment to the site of challenge, local cytokine responses in skin-draining lymph nodes were only reduced by the combined application of H_1_- and H_4_-receptor antagonists and mast cell counts remained altogether unchanged by either H_1_R-, H_4_R- or combined antagonism.

**Conclusion:**

Our model demonstrates a role for H_1_- and H_4_-receptors in Th2 cell infiltration and cytokine secretion in allergic skin diseases and suggests further studies to evaluate these findings for therapeutic approaches.

## Introduction

Animal and human studies have demonstrated elevated histamine levels in atopic dermatitis (AD). Histamine is a central mediator in the complex signalling network that leads to the development and maintenance of pruritus [1]. Yet, pruritus in patients suffering with AD, contrary to the effects of anti-histamines observed in patients with pruritus in allergic rhinoconjunctivitis, is often not relieved by antihistamines [2] which led to the assumption that histamine is binding to other histamine receptors, possibly expressed on the immune cells involved in AD. The H_4_R is expressed on different immune cells [3] and has thus been a focus of recent attention, as efficient targeting of this receptor is believed to be a promising approach for pruritus but also the inflammatory changes observed in AD. In this line, studies could show that patients with AD express increased levels of H_4_R on T-cells of the peripheral blood [4]. Moreover, Dunford et al. demonstrate that the H_4_R is involved in pruritic responses in mice to a greater extent than the H_1_R [5] and Ohsawa et al. could demonstrate a potent anti-inflammatory effect of combined administration of H_1_R and H_4_R antagonists in a mouse model of atopic dermatitis [6]. However, there have also been contradictory studies. For example, H_1_R or H_4_R antagonists had no impact on the development of acute skin lesions in an experimental canine atopic dermatitis model [7].

Skin contains around 20 billion T-cells in humans [8] which conduct immunosurveillance and are associated with the development of inflammatory disorders such as atopic dermatitis [9]. Amongst those T cells are antigen-specific T-helper (Th) subsets with different roles. The T-cell response in AD is biphasic with an initial phase predominated by Th2 cells and a chronic Th1-dominated phase [10]. A number of animal models have been published which allow studies on the role of specific mediators in the skin's immune homeostasis and pathogenesis of AD [11]. The beneficial effects of a combined H_1_R and H_4_R application on pruritus have been demonstrated in such models [6], [12]. However, the role of antigen-specific T-cell subsets cannot be specifically addressed in these models, as tracking of antigen-specific T-cells is not possible in polyclonal models. Studies which clarify the role of the H_4_R for antigen-specific Th2-mediated pathology in AD could emphasize their utility in the treatment of AD. In the study presented below, we describe the development of a murine model of Th2-dependent antigen-dependent skin inflammation which we utilized to demonstrate differential effects of the H_1_Rs and H_4_Rs on Th2 cell migration and cytokine secretion.

## Materials and Methods

### Animals

Six to eight week-old female BALB/c mice were purchased from Charles River Laboratory (Charles River) and housed in the animal facility of the Hannover medical school. DO11.10 (BALB/c-Tg(DO11.10)10Loh/J) mice on a BALB/c background with OVA-specific transgenic (Tg) TCR were bred in our facility. All experimental methods described in this manuscript were in accordance with the German Animal Welfare Legislation and performed as approved by the Lower Saxony State Office for Consumer Protection and Food Safety (LAVES; application no. 33.9-42502-04-09/1664). Animal treatments (patching, intranasal application) were performed under isoflurane anesthesia, and all efforts were made to minimize suffering.

### Generation of polarized T-cells and *in-vitro* restimulation

CD4^+^ T cells were isolated from the spleens of BALB/c or transgenic mice by negative selection using lab grown antibodys (Ab) to MHC class II I-A^d^ (clone 212.A1), CD8 (clone TIB 105), B220 (clone TIB 164), and FcR (clone 24G2) followed by anti-Ig-coated magnetic beads (Polysciences). Syngeneic T cell-depleted splenocytes were used as antigen presenting cells (APC) and were prepared by Ab-mediated rabbit complement lysis using Abs to CD4 (clone GK1.5) and CD8 (clone TIB 105) followed by mitomycin C treatment (Sigma-Aldrich) [13], [14]. 0.5×10^6^/ml CD4^+^ T cells and 1.0×10^6^/ml APCs were cultured with 5 µg/ml pOVA_323–339_ and (i) with 10 U/ml recombinant murine IL-2 (Roche), 10 ng/ml recombinant murine IL-4 (PeproTech), and anti-IFN-gamma (XMG1.2), or (ii) with 25 U/ml recombinant murine IL-2 (Roche), 5 ng/ml recombinant murine IL-12 (Peprotech) and anti-IL-4 (11B11), or (iii) with 20 ng/ml recombinant murine IL-23 (eBioscience), 2 ng/ml recombinant human TGFbeta (Peprotech), 40 ng/ml recombinant murine IL-6 (Miltenyi Biotec), anti-IL-4 (11B11), anti-IL-2 (JES6-1A12) and anti-IFN-gamma (XMG1.2) for 4–7 days to generate (i) Th2 cells, (ii) Th1 cells or (iii) Th17 cells. DO11.10 Th2 purity before transfer ranged between 92–98% as verified by CD4^+^KJ1-26^+^ staining by flow cytometry.

### BMDC preparation

For generation of bone marrow derived dendritic cells, bone marrow cells were isolated by perfusion of femur and tibia. Cells were cultured in RPMI 1640 (Invitrogen Corp.) in the presence of 1% culture supernatant from GM-CSF producing J558L cells [15]. After 6 days, cells were harvested and a fraction of DCs were subjected to FACS analysis for purity control with MHCII/CD11c+ cells constituting between 60–70% of cells analyzed.

### Animal treatment protocols for adoptive transfer and epicutaneous challenge

**Figure 1:**
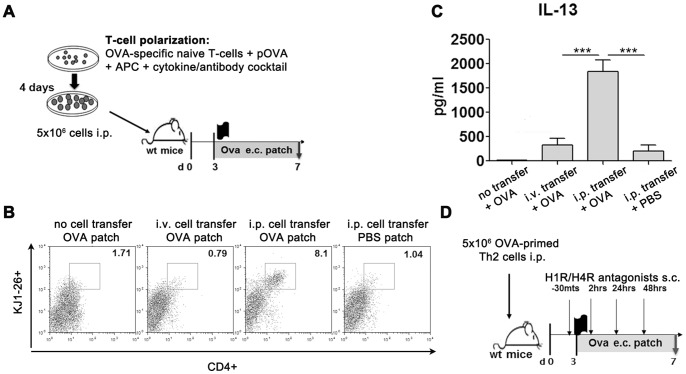
Protocol of adoptive transfer and application of H1R/H4R antagonists. (A) Adoptive transfer protocol. (B) FACS analysis of single-cell suspensions of patched skin from BALB/c recipient mice reveals infiltration of transferred cells into antigen-patched skin after intraperitoneal (i.p.) transfer but not after intravenous (i.v.) transfer. Lymphocytes were identified based on their FSC/SSC characteristics. Depicted are flow cytometric stainings for KJ1-26/CD4^+^ cells identifying transgenic OVA-specific T-cells. Dot plots are representative examples of n = 1 animals with n = 4 animals/group. (B) Supernatant analysis after OVA-specific restimulation of skin dLNs for 3 days *via* ELISA reveals induction of significant levels of IL-13 after intraperitoneal transfer while intravenous transfer fails to elicit significant IL-13 secretion. (D) Adoptive transfer protocol for histamine antagonist studies. ***p<0.001. Columns and error bars represent mean ± SEM of n = 4 animals/group, experiments were performed independently 2 times, analyzed separately and pooled for graphic depiction. pOVA  =  OVA-peptide, APC  =  T-cell depleted splenocytes.

5×10^6^ *in vitro* differentiated, OVA primed Th2 cells of DO11.10 background were injected intraperitoneally (i.p.) into naïve BALB/c mice. One day later, the back of the recipient mice was shaved with electric clippers and two days later, they were exposed epicutaneously (e.c.) to an occlusive patch (Hansaplast), fixed with an adhesive gel (Mastisol liquid adhesive, Eloquest) containing 100 µg OVA (Grade V; Sigma Aldrich) in 40 µl of PBS or PBS alone on shaved back skin as previously described [16]. Patches were left intact for 4 days (Fig. 1A). Intraperiteonal transfer was chosen as preliminary experiments had revealed efficient migration and local induction of cytokine secretion of intraperitoneally transferred Th cells compared to the migration efficiency of i.v. transferred cells (Fig. 1B and C).

### Animal treatment protocol for histamine antagonist administration

For the histamine antagonist experiments, H_1_R antagonist (Mepyramine, Sigma-Aldrich) and/or H_4_R antagonist (JNJ7777120) (gift from Dr. Armin Buschauer, University of Regensburg) were dissolved in 20% (v/v) DMSO and further diluted in PBS. 100 µl of antagonist solution was administered subcutaneously to the site of antigen challenge 30 minutes prior to OVA-challenge as well as 4 hrs, 24 hrs and 48 hrs after the challenge in the same dose (20 mg/kg, 10 mg/kg or 2 mg/kg body weight) [5]. Doses were chosen from previously published studies [7], [17]. Control mice received vehicle treatment. Mice were killed and analyzed on day 7, as preliminary time–kinetic experiments had revealed optimal divergence of read-out parameters in sham-treated vs. antigen-treated animals at this time point (Fig. 1D). For histamine and mouse mastcellprotease-1 measurements animals were analyzed 6 h, 12 h and 24 h after administration of OVA patch.

### Skin preparation

Skin sections (2×2 cm) taken from areas of antigen-/vehicle-exposed skin were pooled from each group, manually minced into fragments by small surgical scissors, and incubated at 37°C for 2 hrs in digestion mix consisting of 450 U/mL collagenase IV (Worthington Biochemical Corporation), 20 µg/mL DNase I Type IV from bovine pancreas (Sigma-Aldrich), 25 mg/ml hyaluronidase (Sigma-Aldrich), 1% HEPES and 1% sodium pyruvate in RPMI medium. After digestion, the tissue was grated through a metal strainer and consecutively through a 100 µm nylon cell strainer (BD) to gain single-cell suspensions. Cells from each group and were cultured with IL-2 (25 U/ml) for 48 hrs prior to harvest of supernatants.

### Lymph node preparation

Axillary lymph nodes (aLNs) were pooled from each group at the time of sacrifice. Single-cell suspensions were obtained by grating through a 100 µm nylon cell strainer (BD). 2×10^5^ cells were cultured with 200 μg/ml OVA presented by GM-CSF differentiated bone marrow–derived dendritic cells (2×10^4^) from wild-type mice for 48 hrs.

### Cytokine ELISA

Cytokines levels of IFN-gamma, IL-4, IL-17 and IL-13 in culture supernatants were determined by means of ELISA DuoSet kits (R&D Systems) according to the manufacturer's instructions.

### Mouse mast cell protease -1 (mMCPT-1) ELISA

Individual sera of mice were subjected to mMCPT-1 ELISA (eBioscience) according to manufacturers' instructions.

### Flow cytometry analysis

All staining procedures were performed on ice. Cells were incubated with anti-FcR (clone 24G2, lab grown) antibody for 20 minutes on ice, then stained with antibodies. The presence of OVA-transgenic T-cells was determined by staining with fluorescently labeled antibodies: CD4 (clone RM4-5; BD) in combination with the anti-DO11.10 TCR antibody clone KJ1-26 (lab grown). The cells were analyzed on a LSRII ﬂow cytometer (BD) or FACS Canto II (BD) in association with FlowJo software (Treestar).

### Skin Histology

Paraffin-embedded skin samples were sectioned (5 µm thick) and stained with hematoxylin and eosin (H&E) or giemsa. Pictures were examined at 200X and 600X magnifications using an Olympus BX51 microscope (Olympus) or at 100x, 200x and 1000x magnifications using a Keyence BZ-9000 microscope (Keyence). Mast cells were counted on giemsa stained sections in a blinded fashion by analyzing 10 high power fields (1000x magnification) per section.

### Mass spectrometry

Skin sections (2×2 cm) taken from areas of antigen-exposed skin were processed individually, weighed and snap-frozen. Prior to histamine analysis in serum and skin tissue, sample preparation was performed as follows: 50 µL of serum were treated with 200 µL of 50/50 acetonitrile/methanol [v/v]. After centrifugation (20,800×g; 10 min; 4°C) supernatant fluid was dried under a constant nitrogen stream. The residual pellet was resolved in 50 µL of 80/20 acetonitrile/water [v/v] containing 0.25 µM d4-histamine as internal standard and analyzed by liquid chromatography coupled with tandem mass spectrometry. Tissue sample preparation was performed using a FastPrep®-24 instrument (MP Biomedicals LLC, Illkirch, France). Skin samples were transferred into a FastPrep® tube (Lysing Matrix A). 800 µL of ice cold extraction solvent (70/30 ethanol/water [v/v]) were added and tissue was homogenized (5.0 m/sec; 2x for 30 sec). Samples were centrifuged (20,800×g; 10 min; 4°C) and 600 µL supernatant fluid were treated as described above. Skin samples were diluted (1∶50) in 80/20 acetonitrile/water [v/v] containing 0.25 µM d4-histamine for analysis.

Mass spectrometric analysis was carried out by a 5500QTRAP^TM^ (AB SCIEX, Massachusetts, USA) equipped with an electro spay ionization source operating in positive ionization mode. Analyte specific mass transitions were m/z 112.2→67.8 for histamine and m/z 116.2→99.1 for d4-histamine. The chromatographic setup consisted of a binary pump system (LC-30AD), a degasser (DGU-20A5), a temperature controlled autosampler (SIL-30AC), a column oven (CTO-20AC) and a control unit (CBM-20A) (Shimadzu, Hannover, Germany). For chromatographic separation an EC 50/2 Nucleodur 100-3 HILIC column connected with a CC 8/3 Nucleodur HILIC 3 µm guard column (Machery-Nagel, Düren, Germany) was used. Mobile phases were 90/5/5 acetonitrile/water/300 mM ammonium acetate (A) and 5/90/5 acetonitrile/water/300 mM ammonium acetate (B), respectively, each containing 0.1% formic acid. Histamine and d4-histamine were eluted at a retention time of 1.6 min under isocratic conditions using a mixture of 80% mobile phase A and 20% mobile phase B at a flow rate of 0.6 mL/min.

### Statistical analysis

Unless indicated otherwise, 4–5 mice were used for each condition studied in an individual experiment. The number of repeat experiments is disclosed in the individual figure legends. For statistical analysis t-tests or one way ANOVA (analysis of variance) with Bonferroni's multiple comparison test was performed with the GraphPad Prism® software to determine statistical differences between means or proportions between two groups of data. A *p*<0.05 was considered to be significant.

## Results

### Adoptively transferred polarized T-cells migrate to the site of antigen-challenge in the skin

To investigate the role of H_1_ and H_4_-receptors in Th2-dependent skin inflammation, we established a model of Th-dependent antigen-specific pathology. To this end, we adapted an adoptive transfer model used to examine allergic lung inflammation [14]. Splenic naïve T-cells derived from DO11.10 mice were subjected to culture conditions for Th2 differentiation. Th polarization status was confirmed by *in vitro* stimulation with PMA and ionomycin (data not shown). Polarized Th2 cells were adoptively transferred into the corresponding recipient mouse strains which was e.c. challenged and analyzed (Fig. 1A).

**Figure 2:**
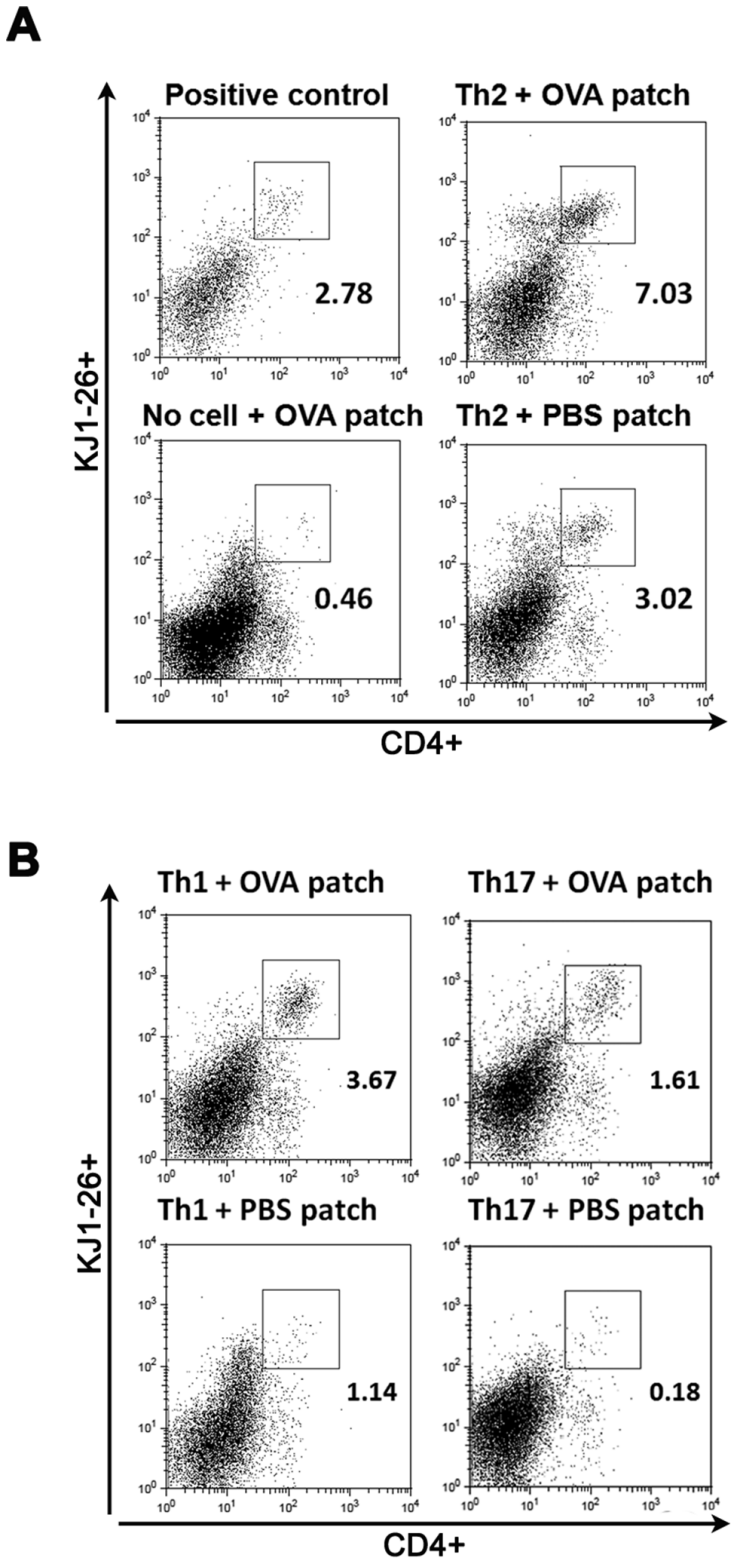
Adoptively transferred polarized T-cells migrate to the site of antigen-challenge in the skin. FACS analysis of single-cell suspensions of patched skin from BALB/c recipient mice reveals increased infiltration of transferred Th2 (A), Th1 or Th17 (B) cells into antigen-patched skin. Lymphocytes were identified based on their FSC/SSC characteristics. Depicted are flow cytometric stainings for KJ1-26+/CD4+ cells identifying OVA-specific transgenic T-cells. “Positive control” refers to skin from DO11.10 mice used to facilitate flow cytometry compensation settings to detect CD4+/KJ1-26+ transgenic T cells. Depicted are representative stainings from one animal/group with n = 4–5 animals/group. Experiments were performed 2–4 times.

Upon flow cytometric analysis of single-cell suspensions from the skin, we could detect increased percentages of the CD4^+^KJ1-26^+^ cells in BALB/c mice which received Th2 cells and were OVA-treated compared to their respective sham-treated controls (Fig. 2A). Sham-treatment of Th2-recipient mice compared to animals which had not received Th2 cells but were antigen-treated also revealed Th2 recruitment (Fig. 2A) suggesting an enhanced skin-migratory propensity of Th2 cells regardless of antigen-contact. Migration of Th2 cells to the site of antigen-contact was neither polarization- nor strain-specific though, as we could also detect increased migration of Th1 and Th17 cells upon OVA treatment (Fig. 2B) and OTII derived Th cells also migrated efficiently to the skin in C57BL/6 mice (data not shown).

### Adoptive transfer of Th2 cells elicits a Th-specific cytokine secretion profile and histological changes

**Figure 3:**
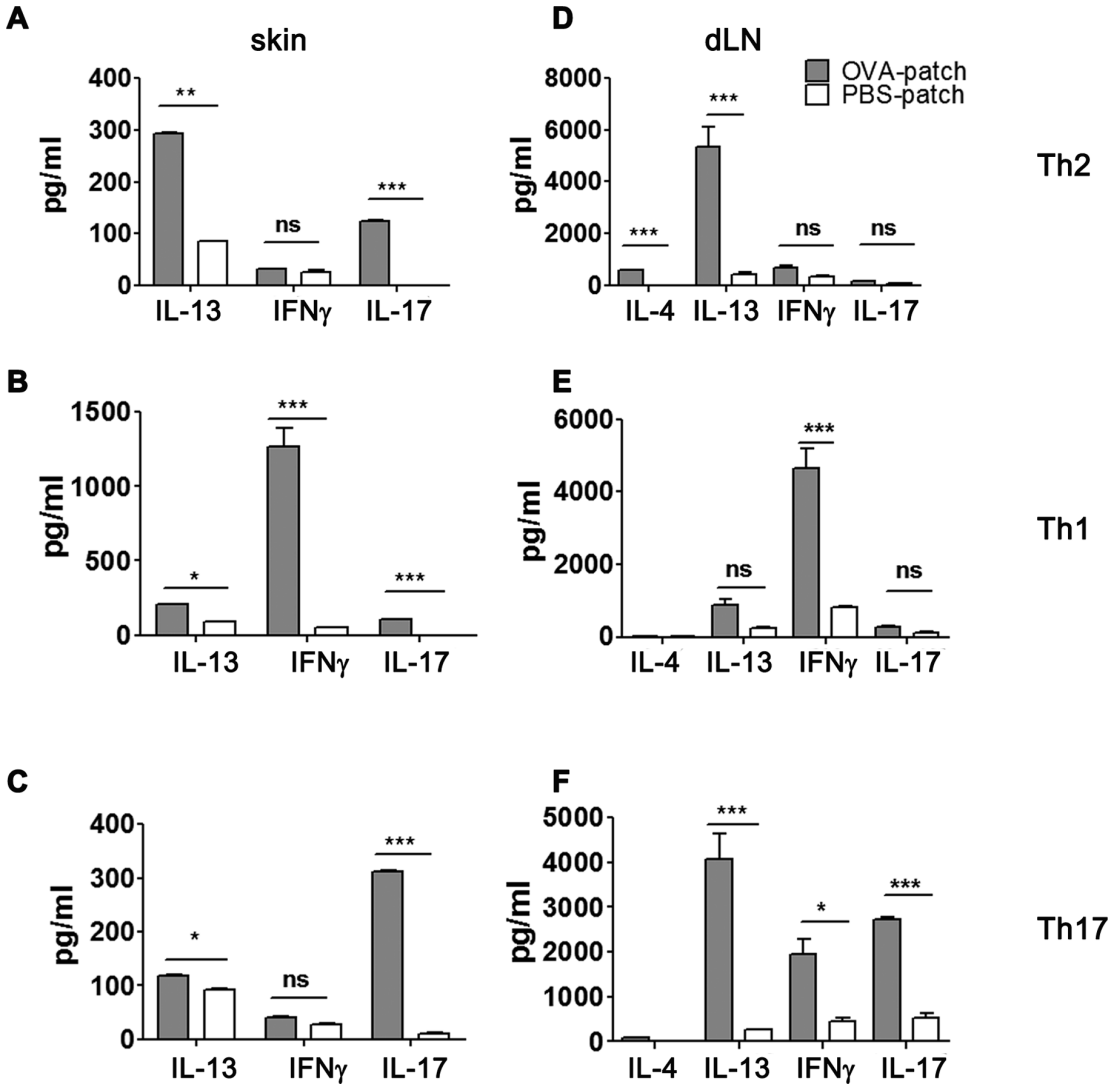
Adoptive transfer of polarized Th cells elicits specific cytokine secretion profiles. (A-C): Supernatant analysis of single-cell suspensions *via* ELISA from patched skin of mice which received Th2 (A), Th1 (B) or Th17 cells (C) after culture with IL-2 (25 U/ml) for 2 days. (D-F): Supernatant analysis of skin dLNs from mice which received Th2 (D), Th1 (E) or Th17 cells (F) after OVA-specific restimulation for 3 days *via* ELISA. Columns and error bars represent mean ± SEM (pooled skin/dLNs (n = 4–5 animals/group) from 3 independent experiments. ***p<0.001; *p<0.05; ns  =  not significant compared to sham–treated control animals.

Having shown that the transferred cells migrate to the site of antigen-challenge, we analyzed the cytokine profile at the site of challenge and in the draining lymph nodes (dLNs) to confirm the local development of a Th2-polarized cytokine milieu. To this end, we stimulated single-cell suspensions obtained from the skin of mice which received Th2 cells and were either OVA- or PBS-patched with IL-2 for 48 hrs. Skin cells from mice which had received Th2 cells and were OVA-treated secreted mainly IL-13 and also small but significant amounts of IL-17. IFN-gamma levels were low regardless of patch-treatment and did not differ significantly between groups. IL-4 levels were consistently below the detection limit in these cultures (Fig. 3A).

In the sham-treated group IFN-gamma levels were low and IL-17 levels were below the detection limit. We observed IL-13 secretion in this group at intermediate levels, suggesting that shaving and patching alone induced a baseline secretion of IL-13 in the sham control group leading to a “skin background” secretion level of IL-13 [16], [18], as also suggested by background levels of IL-13 in animals which had received Th1 or Th17 cells and were sham-treated (Fig. 3B and C).

Upon restimulation of cells from dLNs, we also observed a Th2-polarized cytokine pattern. Cytokine analysis after antigen-specific *in vitro* restimulation revealed that, in line with their initial polarization status before transfer, cells from mice which had received Th2 cells secreted significant levels of IL-4 and IL-13 while IFN-gamma and IL-17 levels were negligible and did not differ significantly from sham-treated controls (Fig. 3D). Sham-treated mice showed low to undetectable cytokine levels for all cytokines assayed (Fig. 3D).

Experiments with Th1 and Th17 polarized cells confirmed cytokine secretion patterns specific to the transferred cells in dLN (Fig. 3E and F) and skin (Fig. 3B and C). However, again we observed background secretion of IL-13 in the skin for both Th1- and Th17-recipients and background secretion of IFN-gamma and IL-13 for Th17-recipients in the dLNs. In summary, these results suggest this model to be well-suited to study the effects of pharmacological targets, such as the histamine receptors on Th cell-specific driven skin inflammation.

**Figure 4:**
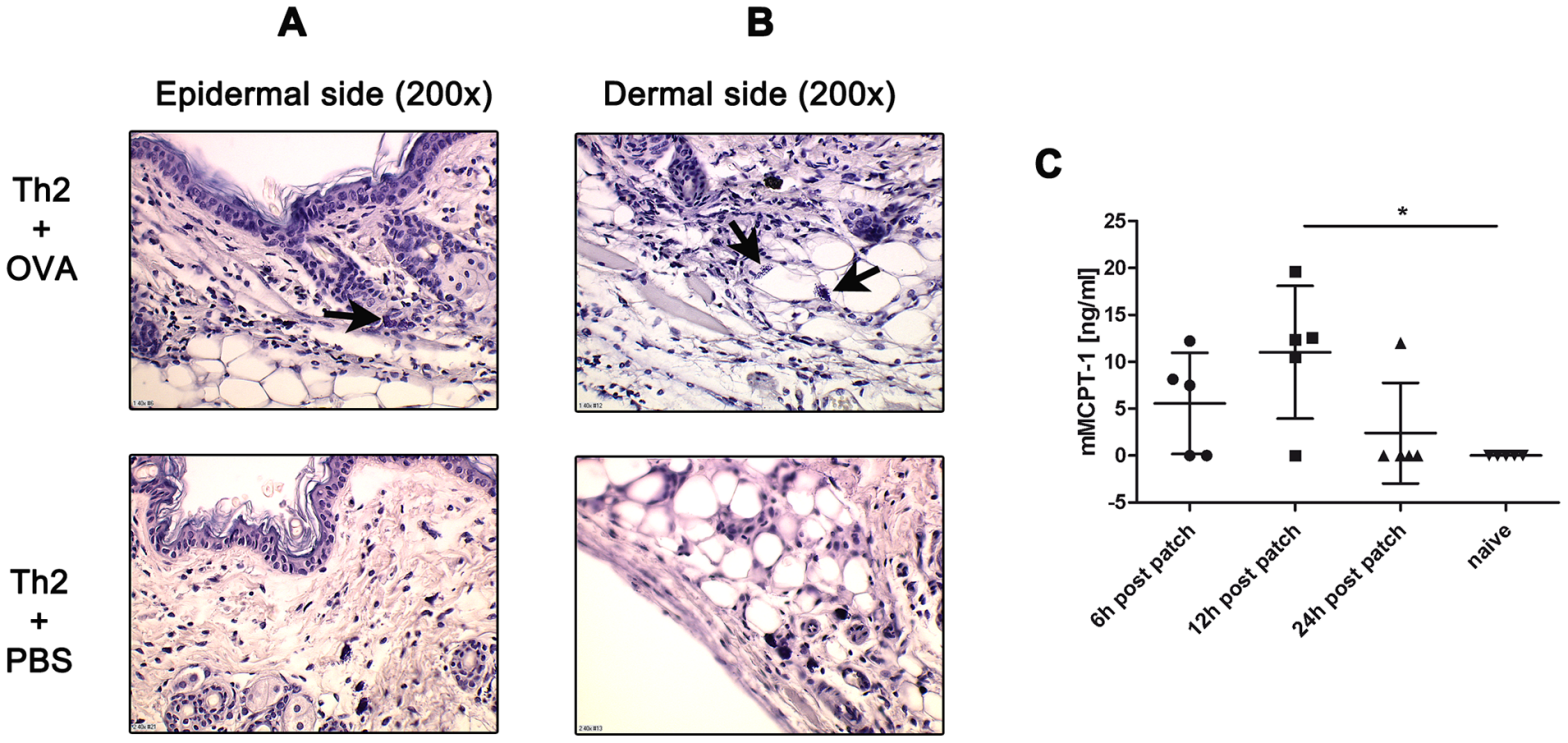
Adoptive transfer of Th2 cells leads to histological changes in skin. H&E staining of epidermal (A) and dermal (B) sides of skin from mice which received Th2 cells and were OVA-treated compared to sham-treated controls. Arrows in A and B indicate degranulated mast cells, magnification 200x. Photos are representative examples of n = 1 where n = 4–5 animals/group were analyzed. All experiments were performed 2–3 times independently. (C) Measurement of mouse mast cell protease-1 by ELISA in sera from individual mice subjected to Th2 transfer 6 h, 12 h or 24 h after OVA patch. Material from naïve mice served as negative control. n = 5 mice/group; experiment performed once. *p<0.05.

Additionally, we performed H&E stainings of histological sections to examine phenotypical changes of the antigen-challenged skin of Th2-recipient mice. The sections showed an increased inflammatory cell infiltration in the epidermis compared to the sham-treated controls (Fig. 4A). Moreover, we detected increased numbers of degranulated mast cells both on the epidermal and dermal side of skin sections of Th2-recipient OVA-treated mice in comparison to the sham-treated groups (Fig. 4A and B). Finally, ELISA measurement of mouse mast cell protease-1 levels as a product of mast cell degranulation in sera of the same mice, revealed an increase in this mast cell product, peaking at 12 h post patching suggesting that mast cells not only increased in numbers but also degranulated (Fig. 4 C).

We thus conclude that in our model migration of transferred Th2 cells is associated with an increased inflammatory cell influx into the skin, particularly mast cells which show evidence for degranulation.

### H_1_- and H_4_-receptor antagonists decrease Th2 migration and cytokine secretion but not mast cell numbers in the skin

**Figure 5:**
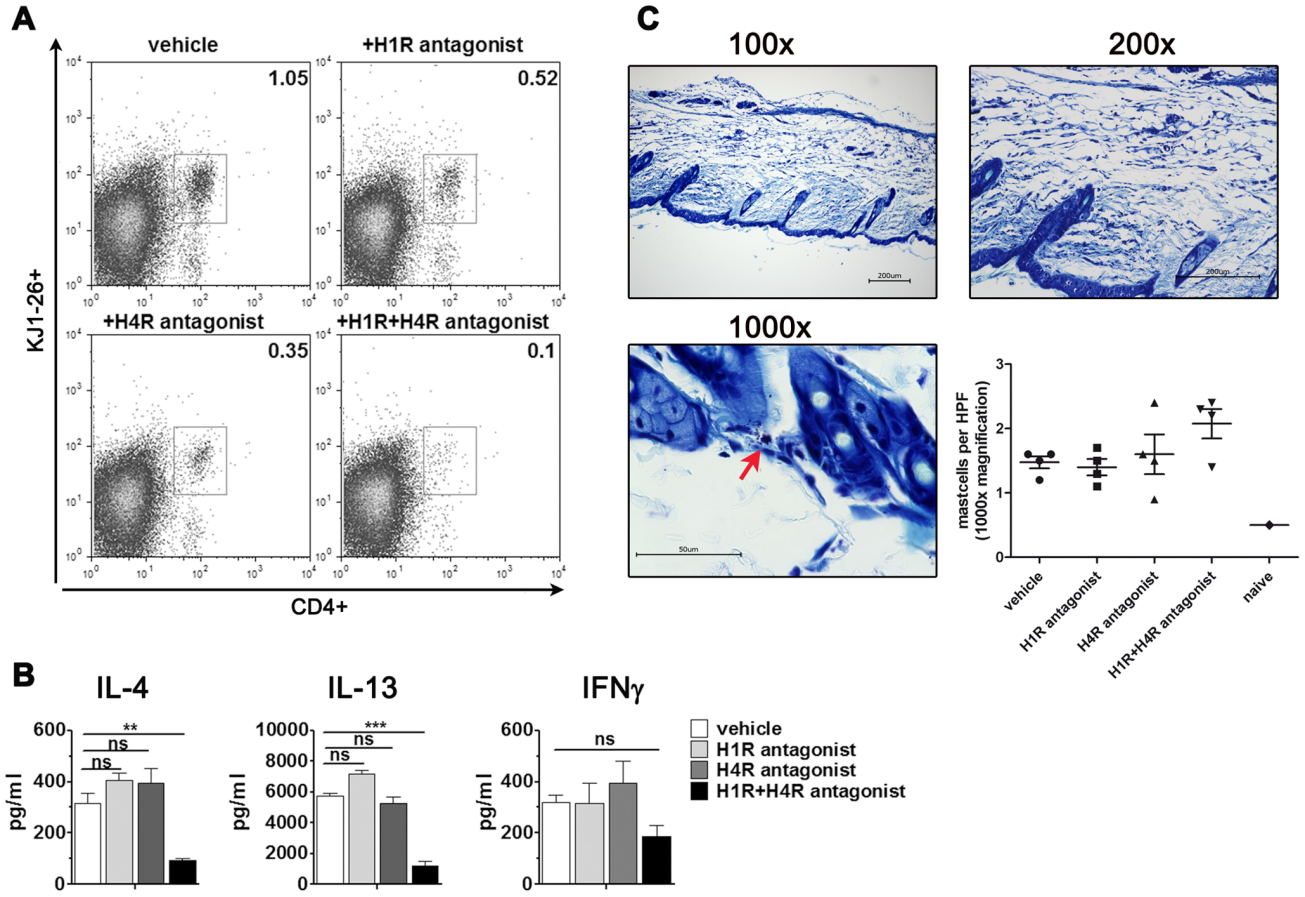
Effect of H_1_R and H_4_R antagonists on Th2 migration, cytokine secretion and skin inflammation. (A) FACS analysis of single-cell suspensions from patched skin reveals reduced infiltration of transgenic T-cells into the skin after treatment with H_1_R and/or H_4_R antagonists (20 mg/kg body weight) compared to vehicle-treated controls. Lymphocytes were gated based on their FSC/SSC characteristics. Depicted are flow cytometric analyses for CD4 and KJ1-26 expression, identifying transgenic OVA-specific T-cells. Experiments were performed independently 2 times; shown are representative stainings of pooled samples, n = 4–5 animals/group. (B) ELISA supernatant analysis for IL-4, IL-13 and IFN-gamma secretion from cells from skin dLNs after OVA-specific restimulation for 3 days compared to vehicle-treated control animals. Columns and error bars represent the mean ± SEM from 2 independent experiments with pooled dLNs (n = 4–5 animals/group) being analyzed. (C) Representative giemsa staining of skin from mice which received Th2 cells, were OVA-patched and treated with H_1_R antagonist (20 mg/kg bodyweight) in different magnifications. Arrow indicates mast cell. Scale bar  = 200 μm or 50 µm. mast cells enumerated on 10 HPF per section, n = 4 animals/group. ***p<0.001; **p<0.01; ns  =  not significant.

Having confirmed that our model is suitable to study Th2-dependent skin pathology, we strove to dissect the role of H_1_R and H_4_R on Th2 cell migration in our model. To this end, Th2 recipient mice were treated with the H_1_R antagonist mepyramine and/or the H_4_R antagonists JNJ7777120 prior and after e.c. challenge with OVA (Fig. 1D). Flow cytometric analysis of single-cell suspensions from the challenged skin revealed decreased percentages of CD4^+^KJ1-26^+^ transferred cells after either H_1_R or H_4_R antagonist treatment in comparison to the sham-treated group. The percentage of transferred cells in skin further declined upon administration of both the antagonists (Fig. 5A).

We also assessed the antigen-specific cytokine secretion pattern in the dLNs. IL-4 and IL-13 secretion were unaffected in cells from the dLNs of mice which had been treated with either one of the antagonists. However, we observed significantly decreased IL-4 and IL-13 secretion from cells from the H_1_R and H_4_R combined treatment group (Fig. 5B). We did not observe statistical changes in IFN-gamma secretion upon combined administration of both antagonists compared to vehicle treatment (Fig. 5B).

Furthermore, we performed giemsa stainings, a method widely accepted for identification of mast cells [19], of the antigen-challenged skin to examine the effect of histamine receptor antagonism on mast cell numbers. Mast cell counts in the skin of mice subjected to Th2 transfer and OVA patch were increased compared to a naïve controls, but we did not observe changes in mast cell counts due to histamine receptor antagonist treatment (Fig. 5C).

**Figure 6:**
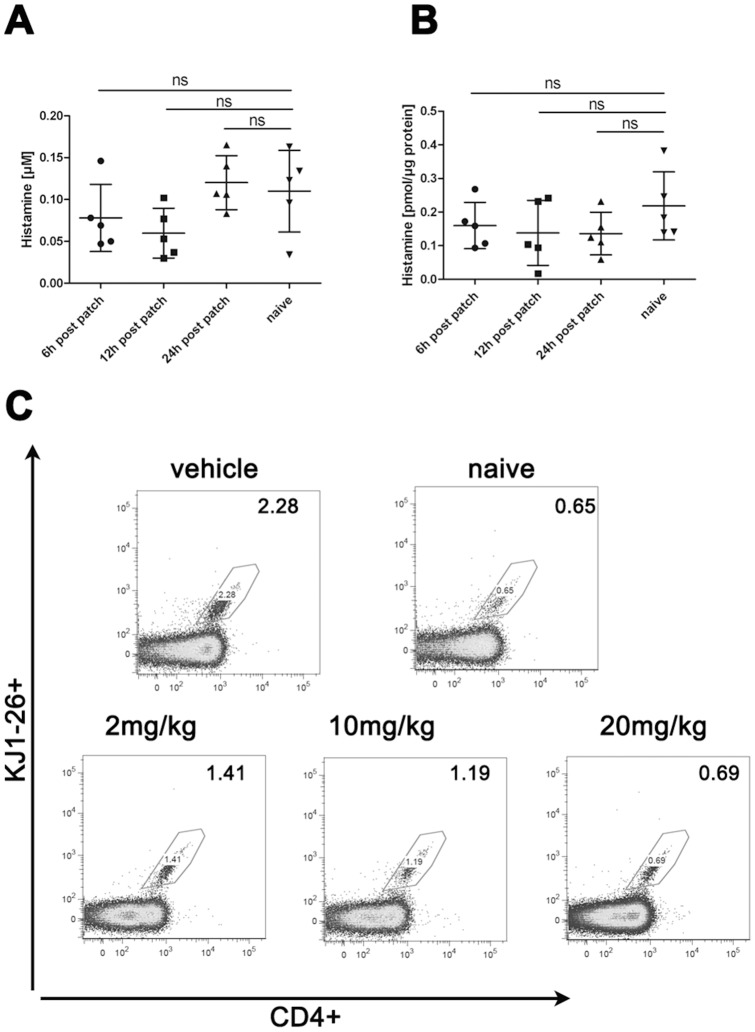
Dose-dependent effect of H4R antagonist and mast cell mediators in skin and serum. Histamine quantification by means of mass spectrometry in sera (A) and patched skin (B) from individual mice subjected to Th2 transfer 6 h, 12 h or 24 h after OVA patch. Material from naïve mice served as negative control. n = 5 mice/group; experiment performed once. (B) FACS analysis of single-cell suspensions from patched skin reveals dose-dependent reduced infiltration of transgenic T-cells into the skin after treatment with H_4_R antagonist (2,10 or 20 mg/kg body weight) compared to vehicle-treated and naïve controls. Lymphocytes were gated based on their FSC/SSC characteristics. Depicted are flow cytometric analyses for CD4 and KJ1-26 expression, identifying transgenic OVA-specific T-cells (pooled skin cells (3–4 animals/group); experiment performed once.

In order to strengthen our conclusion that the observed effects on Th2 migration are dependent on histamine receptor antagonism, we performed two additional sets of experiments: Firstly, to assure that the observed effects of histamine receptor antagonism were due to pertinent histamine levels acting on its receptors, we quantified histamine in serum and skin samples of mice, which received Th2 cells, 6 h, 12 h and 24 h after application of OVA patch by means of mass spectrometry. Indeed we were able to detect significant levels of histamine in our skin samples suggesting that the histamine receptor antagonists could act upon significant levels of this mediator in our model. Albeit in comparison to histamine levels in naïve mice, we found no significant difference in serum or skin from treated mice at the indicated times (Fig. 6 A, B). Secondly, we repeated our experiments with different doses of H4R or H1R antagonists and compared the influx of Th2 cell via FACS analysis with skin from vehicle treated and naïve animals. The 20 mg/kg dose of H4R antagonist, which was used in the previous experiments led to a strong decrease in the migration of transferred T cells to the skin (0.69%) compared to vehicle treatment (2.28%), with the percentage reaching background level as measured in naïve skin (0.65%; Fig. 6C). Reduction of the antagonist dose led to a step-wise recovery of T cell migration to the skin (1.19% and 1.41%; Fig. 6C). In contrast we did not observe any dose-dependent effects by H1R antagonism (data not shown).

Thus, we can conclude that our model allowed us to demonstrate a reduction of Th2 migration to the skin and Th2-dependent cytokine secretion by histamine receptor antagonism. Antagonism of either the H_1_R or the H_4_R alone affected Th2 migration with an additive effect observed upon application of both antagonists. Cytokine secretion and mast cells counts, on the other hand side, were unaffected by H_1_R or H_4_R antagonism alone. Only upon combined receptor antagonism, could we demonstrate effects on Th2 cytokine secretion while mast cell counts remained unaffected by this measure.

## Discussion

There are many models to study different aspects of allergic skin inflammation as a proxy for AD, yet so far neither of these models allowed us to specifically address the role of Th polarized antigen-specific T-cell subsets [11]. Our aim was to establish such a murine model to dissect the contribution of H_1_R- and H_4_Rs to Th2 cell-mediated skin inflammation. In that line, we demonstrate that the combined action of H_1_R and H_4_R antagonists has additive effects on Th2 migration to the skin. Furthermore, we show that the combined application of H_1_R and H_4_R antagonists modulates local Th2 cytokine secretion.

The H_1_R is expressed on diverse cell types and the H_4_R predominantly on keratinocytes and haematopoetic cells [3]. A role for the H_1_R in Th2 cell migration into the lung has been demonstrated in a mouse model of pulmonary inflammation [20]. Different studies have investigated the combined role of H_1_R and H_4_R antagonist in skin inflammation. Combined administration of the antagonists attenuated pruritic and inflammatory responses in different models of skin inflammation and allergic contact dermatitis [6], [12]. However, neither of these studies specifically addressed the contribution of these receptors to Th2 migration to the skin, a question made feasible to address by establishment of an adoptive transfer model only. Our results show for the first time that blockade of either, the H_1_R or the H_4_R, leads to a reduction of H1R- and H4R-dependent migration of antigen-specific Th2-cells to the skin, accompanied by a decrease in the Th2-specific cytokines IL-4 and IL-13 upon combined action of both receptor antagonists.

Several studies support our observations and provide concepts how H_1_R and H_4_R antagonism might act on a cellular level for example by blocking the signals on DCs necessary for Th2 polarization (H1R antagonism) [21] or inhibiting DC chemotaxis (H4R antagonism) [22]. The use of H_4_R antagonists can significantly decrease scratching in mice [23]. Thus in our model, administration of an H_4_R antagonist could lead to a decrease in scratching and in turn, decreased abrasion of the skin, reducing secretion of Th2 cytokines induced by this mechanism [24], thus breaking a vicious cycle. Additionally, decreases in Th2 migration by H_4_R antagonism, as demonstrated by our results, would further dampen Th2 cytokine secretion. Moreover, studies have shown an increased Th2 polarization and increased Langerhans cell activation by H_4_R stimulation [4], [25]. Hence, combined blockade of the H_1_R and H_4_R affects Th2 polarized skin inflammation at several check-points, leading to a more effective reduction in Th2 cytokine secretion than either antagonist alone which is supported by our results.

The lack of effectiveness of classical H_1_R and H_2_R antihistamines in alleviating chronic allergic skin conditions has led the focus of research for antipruritic drugs to shift from histamine to other putative mediators. Although more work is necessary, our results suggest a beneficial effect of a combined H_1_R and H_4_R blockade for the treatment of such conditions. Thus, the development of a new adoptive transfer system for allergic skin inflammation has allowed us to discover previously unappreciated effects of long-standing pharmacological inhibitors, underscoring the usefulness of our model, which will allow us to address other important questions regarding the role of Th cells in allergic skin disease in the near future.

Whilst our model demonstrates increased mast cell counts and mast cell protease-1 levels after Th2 transfer and antigen challenge suggesting increased mast cell degranulation, levels of histamine, the hallmark of mast cell degranulation were not further increased by Th2 transfer and antigen patching compared to naïve controls. This could be due to several reasons. Although measurement of increased histamine levels in serum is an accepted parameter for anaphylactic reactions for example in murine models of food allergy [26], it is less often used in models of skin inflammation. Imai et al. describe increased blood histamine levels in transgenic mice spontaneously developing long-lasting dermatitis (by skin-specific IL-33 expression) [27], a phenotype which resembles the chronic phase of atopic dermatitis. Other groups could show elevated histamine levels in skin or serum only after repeated epicutaneous challenge [28], [29], suggesting that re-exposure might be necessary to induce measurable increases in histamine levels. It is thus conceivable that repeated patching could also lead to histamine increases in our model, a read-out parameter we did not address, though. Thus, the histamine receptor antagonists we employed seem to exert their functions on the background histamine levels we detected in the skin irrespective of patching.

We also did not observe effects of the histamine receptor antagonists on mast cell counts. This aspect, however is not altogether surprising, as we were blocking effects downstream of mast cell infiltration.

Apart from our findings on the contribution of different histamine-receptors in Th2-dependent allergic skin inflammation, our model provided us with additional interesting insights into Th-dependent allergic skin pathology. Our model, which we adapted from widely-used adoptive transfer models for allergic lung inflammation [14], allows tracking of the transferred cells in the patched skin regardless of the strain background, similar to what has enabled researchers in the past decades to dissect the role of different T-cell subsets in analogous lung models. We were also able to demonstrate that the transferred cells induce a distinct cytokine secretion profile in the recipient, largely corresponding to the transferred T-cell subtype. Interestingly, we observed detectable levels of IL-13 regardless of the transfer, polarization status or skin challenge of the mice. These results are in line with other studies which suggest that shaving and patching alone elicit a background level of IL-13 secretion from endogenous skin cells [30], underscoring the critical role of IL-13 in inflammatory skin disease [16]. The additional increase observed in the Th2 recipient OVA treated group most likely results from secretion of the transferred cells which is efficiently abrogated by combined application of the H_1_R and H_4_R antagonists. Upon Th17 cell transfer, we not only observe the expected IL-17 but also significant amounts of IFN-gamma. Prior to transfer, upon *in vitro* restimulation of the *in vitro* generated Th17 cells, we can detect secretion of IFN-gamma as well as IL-17 (data not shown). Hence, after transfer IFN-gamma secretion is possibly due to these transferred cells. IFN-gamma appears to be the most “exclusive” cytokine in the skin as significant increases were only detectable in the mice which had received Th1 cells and were antigen-treated. Taken together, we speculate that the distinct “Th specific” cytokine profiles we observe reflect cytokine secretion by the transferred cells and the “background secretion” of IL-13 and IL-17 is provided by endogenous cells; a hypothesis where future studies in our model will be able to make contributions towards an increased understanding of the contribution of different Th subsets in antigen-specific skin inflammation. Hence we conclude that our model warrants further investigation with regards to the cytokine profiles and histological patterns associated with Th1- and Th17-transfer.

In summary, in addition to the development of an interesting new tool for future experiments to dissect the role of different Th subsets and their mediators in allergic skin disease, our results show that the combined treatment with H1R antagonist and H4R antagonist is superior to treatment with single antagonists in reducing the migration of antigen-specific Th2 cells to skin, as well as reducing Th2-dependent cytokine secretion. These findings corroborate recent results from other groups describing a similar effect on pruritus and skin pathology in models of chronic dermatitis and allergic contact dermatitis [6], [31]. Furthermore, a similar conclusion has been drawn in the study of Beermann et al. [32] who investigated the role of H_1_R and H_4_R antagonists in a model of allergen-induced asthma. Together, we and others provide evidence that the combined treatment approach is a promising new venue for the treatment of different types of allergic diseases.
